# Sexual difficulties in old age and Person-Centered Therapy: A qualitative study with older adults

**DOI:** 10.1192/j.eurpsy.2022.1662

**Published:** 2022-09-01

**Authors:** S. Von Humboldt, J.A. Ribeiro-Gonçalves, G. Low, I. Leal

**Affiliations:** 1 ISPA – Instituto Universitário, Lisbon, Portugal, William James Research Center, Lisbon, Portugal; 2 University of Alberta, Faculty Of Nursing, Edmonton, Canada

**Keywords:** Sexual desire, sexual difficulties, person-centered therapy, Older Adults

## Abstract

**Introduction:**

Sexual well-being (SWB) of the older population can be significantly influenced by age (1) and sexual difficulties (2).

**Objectives:**

Through qualitative research, this study focused on sexual themes that affect the SWB addressed by the older people in person-centered therapy.

**Methods:**

Twenty-five older adults, aged between 65 and 82 years and residents on the community participated in this study.

**Results:**

The results revealed eight main themes for these participants: Absence of a partner, family interference, dissatisfaction with the body, cleanliness and body care, problems in sexual function, physical violence, problems in sexual communication and fear of contracting sexually transmitted diseases. The most discussed themes were the absence of a partner, problems with sexual function and dissatisfaction with the body.

**Conclusions:**

This study highlights the importance of exploring the sexual difficulties that the older population feels in relation to their SWB. 1.von Humboldt S et al. Sexual expression in old age: How older adults from different cultures express sexually? Sex Res Social Policy. 2020;1-15. 2.von Humboldt S et al. Are older adults satisfied with their sexuality? Outcomes from a cross-cultural study. Educ Gerontol. 2020;46:284-293.

**Disclosure:**

No significant relationships.

